# Extracellular Vesicles Tune the Immune System in Renal Disease: A Focus on Systemic Lupus Erythematosus, Antiphospholipid Syndrome, Thrombotic Microangiopathy and ANCA-Vasculitis

**DOI:** 10.3390/ijms22084194

**Published:** 2021-04-18

**Authors:** Martina Mazzariol, Giovanni Camussi, Maria Felice Brizzi

**Affiliations:** Department of Medical Sciences, University of Turin, Corso Dogliotti 14, 10126 Turin, Italy; marti.mazzariol@gmail.com (M.M.); giovanni.camussi@unito.it (G.C.)

**Keywords:** renal disease, autoimmune diseases, HUS, TTP, APS, antiphospholipid syndrome, vasculitis, systemic lupus erythematosus, lupus nephritis, pathogenesis, microparticles, extracellular vesicles, microvesicles, exosomes

## Abstract

Extracellular vesicles (EV) are microparticles released in biological fluids by different cell types, both in physiological and pathological conditions. Owing to their ability to carry and transfer biomolecules, EV are mediators of cell-to-cell communication and are involved in the pathogenesis of several diseases. The ability of EV to modulate the immune system, the coagulation cascade, the angiogenetic process, and to drive endothelial dysfunction plays a crucial role in the pathophysiology of both autoimmune and renal diseases. Recent studies have demonstrated the involvement of EV in the control of renal homeostasis by acting as intercellular signaling molecules, mediators of inflammation and tissue regeneration. Moreover, circulating EV and urinary EV secreted by renal cells have been investigated as potential early biomarkers of renal injury. In the present review, we discuss the recent findings on the involvement of EV in autoimmunity and in renal intercellular communication. We focused on EV-mediated interaction between the immune system and the kidney in autoimmune diseases displaying common renal damage, such as antiphospholipid syndrome, systemic lupus erythematosus, thrombotic microangiopathy, and vasculitis. Although further studies are needed to extend our knowledge on EV in renal pathology, a deeper investigation of the impact of EV in kidney autoimmune diseases may also provide insight into renal biological processes. Furthermore, EV may represent promising biomarkers of renal diseases with potential future applications as diagnostic and therapeutic tools.

## 1. Introduction

Extracellular vesicles (EV) are extracellular structures bounded by a phospholipid bilayer and released by different cell types in biological fluids (blood, urine, synovial fluids) through various mechanisms. First described as “platelet dust” by Peter Wolf in 1967, in recent years EV have gained interest as a commonly recognized important player in cell-to-cell communication, both in physiological and pathological conditions. This depends on their ability to transfer their cargo, consisting of proteins, lipids, or nucleic acids. Interestingly, EV have a role in different biological processes including cell proliferation and differentiation, inflammation, immune signaling, angiogenesis, and stress responses [1]. In this review, we will summarize the most recent data on EV biological features and their pathogenic role in immune-associated renal disease, and their potential use as disease biomarkers.

### 1.1. EV Nomenclature

The most recently updated guidelines of the International Society for Extracellular Vesicles (ISEV) recommend using the term “extracellular vesicles” as “the generic term for particles naturally released from the cell that are delimited by a lipid bilayer and cannot replicate” [2]. EV were historically classified into three main subtypes according to their size, biogenesis and biological features: exosomes (the smallest EV generated from the invagination of the endosomal membrane) [1], microvesicles (or EV which are directly released from the plasma membrane) [3] and apoptotic bodies (the largest EV released by apoptotic cells) [4]. However, since a consensus has not yet emerged on specific markers defining each subtype, the ISEV guidelines recommend defining EV according to [2]:

- physical characteristics such as size (small EV and medium/large EV, with ranges defined, for instance, respectively, <100 nm or <200 nm (small), or >200 nm (large and/or medium)) or density (low, middle, high);
- biochemical composition (CD63+/CD81+− EV, Annexin A5-stained EV, etc.);
- descriptions of conditions or cell of origin (podocyte EV, hypoxic EV, large oncosomes, apoptotic bodies) [2].

### 1.2. EV Isolation and Detection

EV can be detected in almost all human fluids (blood samples, cerebrospinal fluid, synovial fluid, urine, bile, saliva, bronchoalveolar fluid) using different techniques depending on the EV population. In the pre-analytical phase, body sample collection, handling, and storage may impact EV concentration, composition, and function. Thus, to follow a preanalytical protocol tailored to the specific body fluid, the cell of origin of EV is crucial [5]. After collection and isolation, EV can be analyzed through various methods. The most commonly exploited and recommended are the following:

- flow cytometry which detects EV passing through a laser beam. Modern flow cytometers may have many lasers and fluorescence detectors, which allow to label them with multiple conjugated antibodies using the same sample. Although widely used, the analysis using flow cytometry has limitations in detecting the smallest EV whose number and surface expression may be underestimated [3]. To circumvent this limitation, an alternative bead-based technique has been developed using specifically activated beads that capture EV with a cocktail of different exosome marker epitopes allowing subsequent simultaneous detection of multiple antigens [6];
- nanoparticle tracking analysis (NTA) which visualizes EV in the liquid phase by light scattering using a light microscope. A video is taken and the NTA software tracks the Brownian motion of individual vesicles and calculates their size and total concentration. NTA with fluorescent mode detects labeled vesicles and provides quantitative and qualitative analysis. NTA can detect vesicles smaller than those distinguished by conventional flow cytometry [3].

### 1.3. EV and Cell-to-Cell Communication

EV are shed into the extracellular environment under physiological and pathological conditions, and their release is increased by cellular stress conditions as inflammation, hypoxia, oxidative stress or infections [7]. Once released into the circulation, EV have a half-life ranging from a few minutes to a few hours, during which they can be taken up from cells by different mechanisms such as endocytosis or fusion with the membrane of the recipient cell [3]. Circulating EV mainly originate from platelets, erythrocytes, leukocytes, and endothelial cells; while urinary EV mainly derive from podocytes, tubular cells, and epithelial cells [8]. EV may transfer to the target cell receptors, allowing cell signaling in cells that are originally devoid of receptors or by enhancing their number. Moreover, EV may release or transfer proteins as cytokines and growth factors translating in the modulation of target cells and their extracellular environment. Furthermore, EV may transfer messenger RNAs (mRNAs) and microRNAs (miRNAs) to target cells, modulating their function or changing their phenotype [3].

## 2. EV in Physiological and Pathological Settings

**Figure 1:**
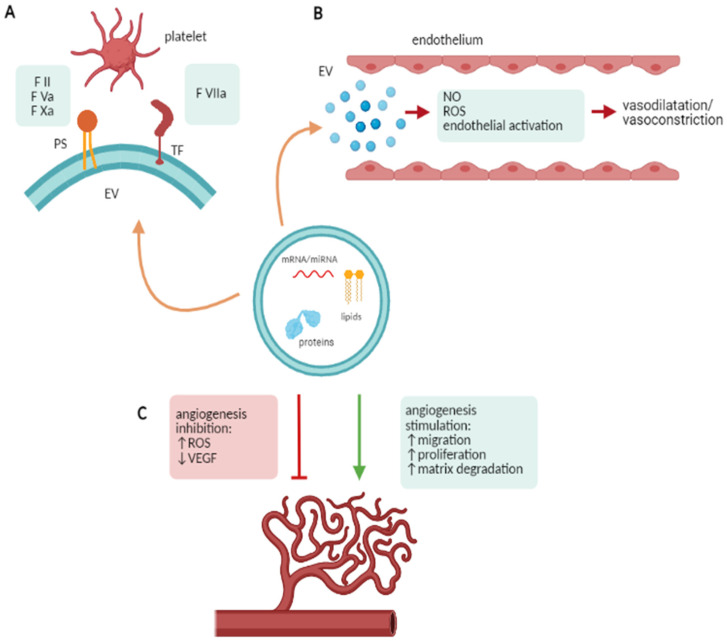
EV in physiological and pathological processes. (**A**) EV and coagulation: EV enriched in phosphatidylserine (PS) and tissue factor (TF) can promote the activation of the coagulation cascade as well as platelet aggregation. (**B**) EV and endothelial activation: EV can modulate vasodilatation and vasoconstriction by activating endothelial cells, releasing cytokines, reducing nitric oxide (NO) production and increasing reactive oxygen species (ROS) production. (**C**) EV and angiogenesis: EV can modulate angiogenesis by increasing ROS production, downregulating the VEGF pathway, modulating the migration and proliferation of endothelial cells and matrix degradation. This figure has been created using Servier Medical Art templates, which are licensed under a Creative Commons Attribution 3.0 Unported License; https://smart.servier.com (accessed on 28 March 2021).

Based on their crucial role in cell-to-cell communication, EV have been involved in several processes such as coagulation and thrombosis, endothelial dysfunction, angiogenesis and immune modulation. Since all of these processes impact renal diseases, a brief discussion will be reported (Figure 1).

### 2.1. EV and Coagulation

Several autoimmune and renal diseases are associated with increased thrombotic risk and thrombotic events. EV are involved in the coagulation cascade as carriers of phosphatidylserine (PS) and tissue factor (TF) and are able to promote the activation of the coagulation cascade. PS can act as a binding site for the coagulation factors II, Va and Xa, enhancing the association between factor Xa and Va and the subsequent conversion of prothrombin to thrombin [9]. TF expressed on the EV surface can activate the extrinsic coagulation pathway by binding factor VIIa [10]. Interestingly, platelet-derived EV have higher pro-coagulant activity compared with activated platelets, possibly due to their enrichment in phosphatidylserine, glycoprotein IIb/IIIa, factor Xa, and P-selectin [11]. Moreover, EV-mediated thrombotic events can be also promoted by their expression of the multimers of von Willebrand factor and stabilization of platelet aggregation (Figure 1A) [9].

### 2.2. EV and Endothelial Dysfunction

Endothelial dysfunction is frequently associated with the pathogenesis of autoimmune diseases: EV contribute to endothelial regulation in physiological and pathological conditions. EV can reduce endothelial and macrophage nitric oxide production, controlling vasodilation. EV can also carry thromboxane A2 acting as vasoconstrictor and platelet aggregation factor [1]. In addition, EV may increase the production of reactive oxygen species (ROS). Finally, as occurs in systemic vasculitis, activated neutrophils release EV expressing the myeloperoxidase (MPO) which by activating the myeloperoxidase-hydrogen peroxide-chloride system, leads to endothelial damage (Figure 1B) [12].

### 2.3. EV and Angiogenesis

Angiogenesis, defined as the growth of new blood vessels from pre-existing ones, is frequently involved in the pathogenesis of autoimmune diseases. EV may be involved in several angiogenesis-associated events [13] as matrix degradation, endothelial progenitor recruitment, and differentiation, migration and proliferation of endothelial cells. Endothelial- and macrophage-derived EV contain pro-angiogenic enzymes (MMP-2, MMP-9, MT1-MMP) which promote extracellular matrix remodeling and favor endothelial cell invasion [14]. On the contrary, lymphocyte-derived EV can inhibit angiogenesis by generating ROS and down-regulating the VEGF pathway (Figure 1C) [15].

### 2.4. EV and Immune System Modulation

**Figure 2:**
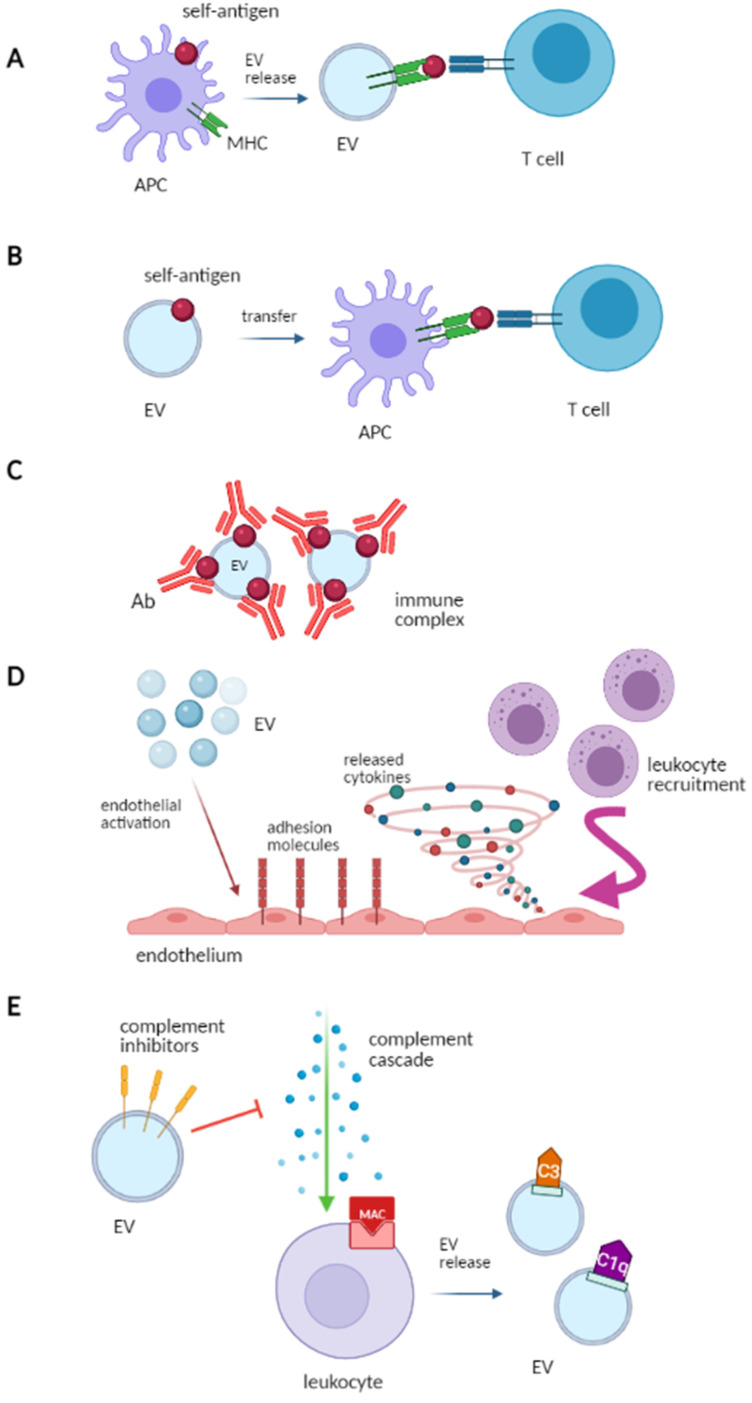
EV and immune system modulation: (**A**) Self-antigen presentation: antigen-presenting cells (APC) can vehicle self-antigens and release extracellular vesicles (EV) which present autoantigens to autoreactive T cells. (**B**) Self-antigen transfer: EV carrying self-antigens transfer them to APC which present them to T cells, triggering an autoimmune response. (**C**) Immune complex formation (IC): EV can participate in IC formation by carrying self-antigens that are bound by circulating autoantibodies. (**D**) Activation of innate immunity: EV can stimulate cytokine release and the upregulation of adhesion molecules on endothelial cells, which favor circulating immune cell recruitment. (**E**) Interaction with the complement system: the complement cascade can be activated on circulating leukocytes which can then release EV exposing complement molecules (e.g., C3, C1q). EV can also convey complement inhibitors and modulate the complement system. This Figure has been created using Servier Medical Art templates, which are licensed under a Creative Commons Attribution 3.0 Unported License; https://smart.servier.com (accessed on 28 March 2021).

EV may affect the immune response by activating innate and adaptive immunity and by participating in the formation of the immune complexes (IC), by acting as autoantigens. Immune cells release immunocompetent EV, which can modulate the immune response by regulating antigen presentation, NK/T cell activation, T cell polarization and immunosuppression (Figure 2) [16].

In the immune system, natural killer (NK) cells have a role in innate immunity, whereas B and T cells are an essential part of adaptive immunity.

- Antigen presentation
  B cells can recognize foreign antigens, while T cells require antigen-presenting cells (APCs) for antigen recognition. Major histocompatibility complex class I (MHC I) and class II (MHC-II) present antigens to CD8+ and CD4+ T cells, thereby activating the immune response. APCs- and B cells-derived EV express the MHC-I, MHC-II and the T-cell costimulatory molecules, thus may take part in the antigen presentation process and in the CD8+ and CD4+ T cell activation (Figure 2A) [17].
- Source of self-antigens and IC formation
  EV participate in the formation of IC. Indeed, EV can express both self-antigens and MHC complexes and may activate autoreactive T-cells in autoimmune disease. As an example, the synovial fluid of patients with rheumatoid arthritis contains IC composed of platelet-derived EV and autoantibodies against citrullinated peptides [18]. Similarly, in systemic lupus erythematosus (SLE), EV carry nuclear molecules, which represent a potential source of autoantigens and participate in IC formation. Furthermore, EV-associated ICs may affect the recognition and clearance of EV by phagocytes, leading to the accumulation of cell debris and triggering the autoimmune response (Figure 2B,C) [19].
- Role of adjuvants in innate immune response
  Leukocyte-derived EV activate the endothelium by upregulating adhesion molecules and releasing cytokines. This leads to leukocyte recruitment via platelet-derived EV, which promotes monocyte adhesion to the endothelium [20]. Dendritic cell-derived EV increase the NK cytotoxic activity and stimulate the release of proinflammatory cytokines by epithelial cells (Figure 2D) [21].
- Role in complement activation
  When the complement system undergoes activation, the membrane attack complex may be set down on blood cells and complement-coated EV may be released. C3-positive EV reflect the activation of the alternative pathway of the complement, while C1q-positive EV reflect the activation of the classic pathway [22]. Moreover, EV may express complement regulators on their surface (complement receptor type 1, membrane cofactor protein, decay-accelerating factor also denoted as CD59), thereby inhibiting the membrane attack complex (Figure 2E) [23].

## 3. EV and Renal Intercellular Communication

Recent studies have shown the relevance of EV in renal intercellular communication. Although their role has not been yet fully understood, their potential application as diagnostic, prognostic, or therapeutic biomarkers for various kidney diseases is currently being explored [8].

In physiological conditions, the glomerular filtration apparatus prevents circulating EV from reaching the lumen of renal nephrons, thus circulating EV may stimulate kidney cells facing the vascular compartment and the immune cells [24]. However, EV are released from renal cells into the renal tubule and can be detected in urine. The release of renal EV may vary during renal disease, thus urinary EV have been proposed as potential biomarkers of kidney injury. Urinary EV are largely composed of EV released from renal cells as confirmed by proteomic analysis. Urinary EV carry specific proteins, mRNAs and miRNAs, which reflect their cellular origin. Indeed, EV from glomerular podocytes contain podocin and podocalyxin [25], EVs from proximal tubular cells express cubilin, megalin, aminopeptidase [26] and aquaporin-1; EVs from the Henle’s loop contain type 2 Na-K-2Cl cotransporter, CD9 and Tamm–Horsfall protein [27]; EVs from collecting ducts carry AQP-2 and mucin-1 [28]. The majority of urinary EV are released from the first part of the renal nephron, with a limited contribution from the lower urinary collecting system [29]. Interestingly, EV may have a role in cell-to-cell communication between the proximal and distal renal tubules. EV released from glomerular podocytes, via the urine flux, reach and can be taken up by the epithelial cells of the distal tubule and the collecting duct [30]. A recent study has investigated a potential hormonal mechanism that regulates EV uptake. It has been demonstrated that in vitro stimulation with desmopressin selectively stimulates the EV uptake in tubular cells, while a vasopressin antagonist reduced in vivo the uptake of EV injected within the renal tissue [31]. Moreover, EV may take part in the regulation of renal inflammation. Indeed, it was reported that EV from proximal tubular cells cultured in the presence of a dopamine receptor antagonist are able to reduce ROS production in distal tubular cells [32]. Interestingly, EV can modulate renal tissue repair and fibrosis after renal damage [33,34,35]. In vitro studies have demonstrated that hypoxic conditions increase the release of tubular cell-derived EV, which induced fibroblast activation and proliferation by the transfer of TGFβ mRNA [36]. EV may also control the ion transport, as EV from proximal tubule cells can decrease ENaC activity in the distal tubule and collective ducts [37]. Moreover, EV can also mediate the transfer of aquaporin 2 from cells of the upper collective duct to those of the lower collective duct, increasing water transport in the recipient cells [38]. Urinary EV may also exert protection against bacterial infection of the urinary tract. As a matter of fact, urinary EV carry antimicrobial peptides and can inhibit the growth of the most common urinary pathogen *Escherichia coli* by inducing their lysis [39]. Mesenchymal-to-epithelial transition is a crucial process in kidney development and regeneration. It has been reported that this process is mediated by tubular epithelial cell-derived EV which carry a specific subset of miRNAs, driving the differentiation of mesenchymal stem cells into epithelial cells [40]. Interestingly, mesenchymal stem cells and endothelial progenitor cells release EV that induce nephron regeneration and repair by promoting tubular proliferation and by inhibiting apoptosis [41,42].

## 4. EV in Renal Disease

### 4.1. Antiphospholipid Syndrome

Antiphospholipid syndrome (APS) is a systemic autoimmune disease characterized by recurrent arterial or venous thrombosis and/or obstetric complications in the presence of antiphospholipid antibodies (aPL). APS is the most common cause of acquired thrombophilia and is associated with decreased survival. A severe form of APS, termed catastrophic antiphospholipid syndrome, occurs in <1% of patients with aPL and is associated with high mortality [43]. Despite the clinical relevance of this syndrome, its pathogenesis is not yet fully understood and recent studies have focused on EV as potential damage mediators.

Various studies have reported an increased level of EV in APS patients. Štok et al. [6] have compared APS patients, patients with idiopathic thrombosis and healthy controls and found that circulating EV were increased in patients with a history of thrombotic events (APS patients and patients with idiopathic thrombosis) compared to healthy subjects, suggesting a chronic cell activation even in the absence of an acute thrombotic event. This study demonstrated the presence of platelet, endothelial cell, lymphocyte, antigen-presenting cell-derived EV in patients with a history of thrombosis as well as in healthy controls. These EV express molecules involved in platelet/endothelial function, immune regulation extracellular matrix regulation and cell-to-cell adhesion. The analysis of EV surface proteins demonstrated an increased expression of CD8, CD44, CD133/1 and CD62P in the aPL patients. The increased expression of CD133/1 and CD62P on the EV surface in APS patients could reflect the increased endothelial and platelet activation, respectively, and their possible contribution to the thrombotic events [6]. Endothelial- and platelet-derived EV were increased in aPL patients, suggesting a chronic activation of endothelial cells and platelets. Interestingly, a correlation between the level of endothelial-derived EV and the level of anti-β2GPI has been demonstrated which closely correlates with thrombosis [44].

Breen et al. [45] have shown an increased level of endothelial- and platelet-derived EV in aPL patients compared with healthy subjects, while no difference between obstetric APS or asymptomatic aPL patients was detected. Moreover, plasma from patients with APS and from patients with SLE aPL+ or SLE aPL- increased the release of EV from cultured endothelial cells compared to the plasma of healthy subjects. Of note, only plasma from APS patients caused the release of EV with significant procoagulant activity [46]. aPL induces tissue factor (TF) synthesis in endothelial cells in vitro, and it has been reported that TF+ EV are elevated in aPL+ patients [44]. In particular, TF expression on endothelial-derived EV is increased in APS patients compared to healthy subjects [47]. Moreover, Willemze et al. [48] have demonstrated that EV from APS patients display a higher TF activity compared to asymptomatic aPL+ patients.

More recently, Mobarrez et al. [19] have compared anti-β2GPI-positive SLE patients, aPL-negative SLE patients and healthy controls. They found that SLE patients are depleted of β2GPI-positive EV when compared to healthy subjects, and their level is particularly low in anti-β2GPI-positive patients. They also found that β2GPI preferentially binds to the phosphatidylserine (PS)-positive EV, thus suggesting that anti-β2GPI antibodies may bind to the β2GPI-PS complexes on EV resulting in the loss of EV β2GPI expression. β2GPI promotes the clearance of PS+ EV, thus the increased number of PS-negative EV may act as a possible source of autoantigens and thus trigger the autoimmune response [19].

Regarding obstetric APS, pregnant women with APS had increased PS+ EV, endoglin+ EV and endothelium-derived EV compared to healthy controls in the first and second trimester of pregnancy. Conversely, in the third trimester, higher levels of TF+ EV and platelet-derived EV can be detected. According to the authors, this finding could reflect the activation of both endothelial cells and platelets during pregnancy. In particular, high-risk APS patients (triple aPL positivity plus vascular thrombosis and/or severe pregnancy complications/placental insufficiency) have higher endoglin+ EV, TF+ EV and platelet derived-EV in all three trimesters, sustaining a major vascular activation. Interestingly, endoglin is expressed by vascular endothelium and by syncytiotrophoblasts and altered levels of soluble endoglin are linked to vascular disorders as pre-eclampsia [49].

In conclusion, patients with aPL have an elevated level of circulating EV, which may reflect a state of systemic vascular activation. EV have been shown to act as procoagulant and proinflammatory mediators, thus their level may correlate with the thrombotic risk. However, at present, a clear relationship between elevated EV levels and thrombotic events is still missing (Table 1).

### 4.2. Systemic Lupus Erythematosus

Systemic lupus erythematosus (SLE) is a systemic autoimmune disease characterized by the production of autoantibodies against nuclear antigens and the deposition of immune complex (IC) leading to systemic inflammation and tissue damage. Lupus nephritis (LN) is one of the most severe organ manifestations and a primary cause of morbidity and mortality [50].

Studies on circulating EV in SLE patients revealed divergent results regarding their circulating level and characterization. EV were found increased in SLE [51,52], or decreased compared to healthy controls [53]. Those different results reflect the lack of standardized methods for EV evaluation and characterization.

In SLE, circulating EV expose chromatin on their surface and may represent a source of nuclear antigens which can bound both the IgG and the complement, resulting in the formation of IC, which correlate with the disease activity and the vascular damage [52,54]. Circulating EV can bind both IgG and IgM to form immune complexes (EV-ICs) and EV-IgG+ were positively correlated with the disease activity. Platelet-derived EV (PEV), mainly PEV-IgG+, stimulated monocytes in vitro changing their phenotype and promoting their inflammatory response [52]. Several studies have supported the role of the deoxyribonuclease DNASE1L3 as a genetic determinant of susceptibility [55,56]. Sisirak et al. [57] have found that DNASE1L3 can digest chromatin in apoptotic cell-derived EV and, in the absence of DNASE1L3, EV-associated DNA may gather in an extracellular environment and promote autoantibody production by autoreactive B cells. DNASE1L3 is produced by dendritic cells and macrophages, and its circulating level was inversely correlated with anti-dsDNA level [57].

Platelet activation plays a key role in the pathogenesis of SLE. They promote T and B cell activation, NETosis, type I IFN production, and dendritic cell activation resulting in systemic organ damage. Platelet-derived EV are the most prevalent circulating EV in healthy subjects, while conflicting results have been reported regarding platelet-derived EV in SLE. Burbano et al. [52] have found an increased number of circulating EV (mainly platelet-derived EV) in patients with SLE compared to healthy controls. However, platelet-derived EV level was unrelated to disease activity measured with SLEDAI score. Lopez et al. [58] have observed an increased level of platelet-, monocyte- and T lymphocyte-derived EV. Interestingly, the authors found that EV level is influenced by the disease activity and is related to the activation status of blood parental cells. They also reported that glucocorticoid therapy may influence EV production and T cell activation, as EV from patients treated with glucocorticoids induced the upregulation of CD25 and the accumulation of IL-10 in T cells [58]. Moreover, circulating platelet-derived EV correlated with endothelial-independent vasodilatation in SLE [59].

Patients with SLE have increased cardiovascular risk due to platelet activation and systemic endothelial activation. EV may be involved in this process, as EV and EV-ICs from SLE patients can activate endothelial cells by increasing the expression of adhesion molecules (CD54, CD102), the production of chemokine (CCL2, CCL5, IL-6) and the adherence of monocytes. EV may also mediate endothelial injury by increasing endothelial permeability through the alteration of cytoskeletal proteins leading to adhesion and migration of monocyte to the inflamed organs [60].

Polymorphonuclear leukocytes (PMNs) are greatly involved in SLE pathogenesis, as PMNs showed generalized hyperactivity, with enhanced apoptosis and increased production of neutrophil extracellular traps (NETs) [61]. Moreover, increased oxidative stress has been observed in SLE, which may contribute to immune dysregulation [62]. Winberg et al. [63] have found that EV from SLE patients induced ROS production in the patient’s own PMNs re-suspended in autologous serum, particularly in patients with low circulating C3 level, which reflects disease activity. The ROS production partly depends on EV properties, serum components (including autoantibodies) and PMN hyper-responsiveness [63].

In vitro studies have shown the proinflammatory effect of EV on blood-derived plasmacytoid dendritic cells and myeloid dendritic cells by increasing the expression of costimulatory molecules (CD40, CD80, CD83, CD86) and the release of proinflammatory cytokines (IL-6, TNF, IFNα). Moreover, EV enhanced the formation of neutrophil extracellular traps (NETs) which may represent a source of nuclear autoantigens thus contributing to the pathogenesis of SLE and renal inflammation [64]. EV from patients with active LN contain a higher level of acetylated chromatin compared to patients with remissive LN, without LN, or healthy controls. Rother et al. [65] have found that the degree of EV acetylated chromatin determines their strength to stimulate neutrophils to form NETs.

According to proteomic and flow cytometry analysis, circulating EV in SLE patients have an increased content of IgG and galectin-3 binding protein (G3BP), a glycoprotein that may contribute to the pathogenesis of SLE. G3BP is induced by type I IFN and exhibits a high binding capacity toward components of the glomerular basement membrane (GBM) [66], including collagen IV, fibronectin and galectin-3. Interestingly, patients suffering from lupus nephritis show a glomerular G3BP/IgG co-localization pattern specifically in the GBM, suggesting the presence of G3BP in the IC delivered either by EV from the circulation or locally formed [67]. In SLE patients, in vitro stimulation of peripheral blood mononuclear cells (PBMCs) with TLR-9 agonist increases the release of EV expressing both G3BP and dsDNA. This was particularly relevant in patients with active LN, compared to healthy donors [68].

Urinary EV may reflect structural damage and renal dysfunction, and they have been investigated as potential LN diagnostic and prognostic biomarkers. Urinary podocyte-derived EV reflecting the glomerular podocyte damage are increased in LN and SLE patients compared to healthy controls. Interestingly, urinary EV level is correlated to the SLE disease activity index (SLEDAI) score, anti-dsDNA antibodies titer, proteinuria and histopathological lesions [69]. A recent study demonstrated that urinary EV expressing the high-mobility group box 1 molecule (HMGB1) are higher in SLE patients with active LN than in those without renal involvement, and correlate with proteinuria. HMGB1 is involved in the pathogenesis of several autoimmune diseases and it may be an important mediator in LN. Indeed, its expression is increased in glomerular endothelium and mesangium, and its blood and urinary level is increased in LN [70].

Several studies have identified EV-derived miRNA as markers of renal damage which can also discriminate active LN [71]; miR-21, miR-150, and miR-29c were correlated to renal fibrosis and could predict the progression to the end-stage renal disease [72,73]. A unique circulating miRNA expression profile was detected in class IV LN [74] and urinary EV-derived miRNA have been proposed as peculiar biomarkers of class IV LN [75].

Recently, Garcia-Vives et al. investigated the miRNA expression profile of urinary EV in proliferative LN as a new potential prognostic biomarker. Patients with clinical responses to therapy are characterized by an increased level of miR-31, miR-107, and miR-135b-5p in urine and in renal tissue (mostly localized in epithelial tubular cells), compared to non-responder patients. In vitro stimulation of tubular epithelial cells with proinflammatory cytokines increases the release of these miRNA, which can be taken up by endothelial cells and mesangial cells in responder patients [76].

Recent studies have evaluated the role of mitochondria in autoimmune diseases. Activated cells can release EV containing mitochondria or free mitochondria which may stimulate immunity. Mobarretz et al. [77] have demonstrated the presence of circulating particles (approximately 3 μm), and among them, a population of large EV carrying mitochondrial molecules (mitoEV) were found that both increased and associated with the disease activity in SLE patients. Furthermore, patients suffering from active LN have higher levels of mitoEV and IgG-coated mitoEV, suggesting that they may contribute to the formation of IC and thus be involved in renal damage [77].

In conclusion, EV may play a crucial role in the pathogenesis of SLE, and particularly in LN-associated renal injury. For these reasons, EV have been proposed as potential biomarkers of disease in SLE patients as well as early biomarkers of renal damage in LN (Table 2).

### 4.3. Thrombotic Microangiopathies

Thrombotic microangiopathies include different diseases characterized by microangiopathic hemolytic anemia and thrombocytopenia also potentially involving the kidney. Thrombotic microangiopathies include Shiga toxin-producing *Escherichia coli* (STEC-HUS) and thrombotic thrombocytopenic purpura (TTP).

Haemolytic uremic syndrome (HUS) is characterized by nonimmune microangiopathic haemolytic anemia, thrombocytopenia and acute kidney injury. Typical HUS is subordinate to Shiga toxin-producing *Escherichia coli* (STEC-HUS) infection, which first colonizes the intestine and produces the toxin which enters the bloodstream and causes renal injury.

In STEC-HUS, EV mainly derive from platelets, monocytes, neutrophils and red blood cells. Ståhl et al. have found EV expressing TF and phosphatidylserine potentially involved in the formation of microthrombi. Moreover, in the acute phase of the disease, circulating EV derived from platelets, monocytes, and neutrophils show deposition of C3 and C9 on their surface. Interestingly, EV also express phosphatidylserine, which activates the coagulation factor V and X, thus enhancing and promoting thrombosis [78]. Those findings may reflect the systemic complement activation and the role of EV in the inflammatory and thrombogenic events in HUS [10].

In vitro experiments showed that whole blood incubated with Shiga-toxin and/or STEC-lipopolysaccharide increased the release of TF-positive EV, C3- and C9-positive EV derived from platelets, monocytes and red blood cells [10,22]. It has been speculated that activated complement factors carried by EV can be transferred to recipient cells, driving cell damage [79].

Since STEC are non-invasive bacteria, a small amount of Shiga toxin is present in the circulation; however, EV may transfer the toxin to the kidneys via peritubular capillaries. EV containing Shiga toxin were found within the kidney into renal cells, and in vivo experiments showed that EV enriched in Shiga toxin can reach renal cells through the glomerular and tubular basement membranes. In vitro studies also demonstrated that EV undergo endocytosis in glomerular endothelial cells, leading to cell damage [80].

Shiga toxin interaction with circulating cells is mediated by two different receptors: the globotriaosylceramide (Gb3) toxin receptor and TLR4. Shiga toxin can be taken up by cells after binding to Gb3 or by cellular uptake of EV carrying the toxin derived from different host cells. EV expressing Shiga toxin are taken up by both Gb3-positive and Gb3-negative recipient cells. However, only Gb3-positive host cells are susceptible to toxin-induced cellular damage, reduced cellular metabolism and protein synthesis [81]. Of note, renal endothelial cells express both Gb3 and TLR4. In vitro experiments using soluble Shiga toxin have shown that TLR4 acts as a Gb3 coreceptor, thus facilitating renal cell injury [79].

TTP is a rare microangiopathic hemolytic anemia in which mutations of vWF protease (ADAMTS13) or autoantibodies against ADAMTS13 lead to the deposition of von Willebrand factor (vWF) multimers within capillaries. Systemic endothelial cell injury and platelet aggregation activate systemic microthrombosis mainly involving the brain and the kidney.

A comparison of circulating EV in TTP patients and healthy subjects has shown that platelet-derived EV are the most represented EV subtype in both groups. Moreover, in TTP patients the platelet-derived EV level is significantly higher, as are EV express markers of platelet activation such as CD62p. Interestingly, platelet-derived EV have a procoagulant activity greater than platelets due to phospholipid expression on their surface which triggers the coagulation cascade [82].

Other studies have found higher levels of platelet-derived and endothelial-derived EV in TTP. Endothelial-derived EV have shown procoagulant and proadhesive roles as they express CD62E (E-selectin), VWF, intercellular adhesion molecule 1 (ICAM-1), platelet endothelial cell adhesion molecules (PECAM-1; CD31) and endoglin (CD105) [83]. Tati et al. [84] have shown that circulating endothelial-derived EV are coated with C3 and C9 and complement activated on platelets and glomerular endothelium. Complement activation may represent an ancillary phenomenon to platelet activation and endothelial injury, and thus may drive the microangiopathic process [84].

Unfortunately, only a few studies have investigated the role of EV in TTP, and future research is needed to better understand the pathogenesis of TTP (Table 3).

### 4.4. ANCA-Associated Vasculitis

Systemic vasculitis consists of different syndromes characterized by blood vessel inflammation and multiple organ involvement. Small vessel vasculitis is often associated with anti-neutrophil cytoplasmic antibodies (ANCA), predominantly IgG autoantibodies directed against neutrophil cytoplasmatic constituents such as proteinase 3 (PR3, named cANCA) and myeloperoxidase (MPO, named pANCA). ANCA-associated vasculitis (AAV) comprises microscopic polyangiitis (MPA), granulomatosis with polyangiitis (Wegener’s) (GPA), and eosinophilic granulomatosis with polyangiitis (Churg-Strauss) (EGPA). AAV can affect small vessels in different organs resulting in pauci-immune glomerulonephritis, vasculitis involving the respiratory tract, and is associated with an increased risk of systemic thrombosis [85]. Despite new treatment options that have recently improved AAV prognosis, early diagnostic and prognostic biomarkers are still an unmet need.

Patients with AAV have an increased risk of thromboembolic events due to the hypercoagulable state [86]. ANCA can induce neutrophil activation, neutrophil degranulation and release of neutrophil extracellular traps (NETs), which are involved in the development of vasculitis lesions [87]. Moreover, complement activation via the alternative pathway is crucial for the development of the disease [88]. In vitro experiments have found that both pANCA IgG and cANCA IgG can stimulate C5a-primed neutrophils to produce TF-expressing EV and TF-expressing NETs which, in turn, promote thrombin generation and the activation of the coagulation cascade [89]. Notably, Mendoza et al. [90] have measured the TF activity of EV and found higher TF activity in patients with AAV and associated venous thromboembolism, compared to patients without thrombotic events [90].

Recent studies have demonstrated a potential role of platelet- and neutrophil-derived EV in the pathogenesis of vasculitis. Polyclonal ANCAs isolated from patients and chimeric PR3–ANCA induces the release of EV from primed neutrophils in vitro. Moreover, these EV are enriched in PR3 and MPO, exhibit thrombin-generating capacity, can bind the endothelium and induce its activation, induce ROS production and the release of proinflammatory cytokines (IL-6, IL-8) [91].

An increased level of circulating platelet-, neutrophil- and endothelial-derived EV in patients with vasculitis has also been found [92]. In particular, a high level of neutrophil-EV during the acute phase of the disease which decreased after steroid treatment has been reported, reflecting the key role of neutrophil activation. Of note, endothelial-EV level correlated with the Birmingham Activity Vasculitis Score (BVAS) and acute phase reactants, suggesting their potential application as disease biomarkers [92].

The kinin system contributes to the inflammatory response and the development of vasculitis. The kinins regulate local blood pressure, promote inflammation, and capillary leakage. The kinins achieve their effect through B2 and B1 receptors. The B2 receptor is constitutively expressed and involved in inflammation and hyperalgesia, while the B1 is upregulated during chronic inflammation (such as vasculitis), and controls neutrophil migration. Kahn et al. [93] have found increased circulating leukocyte-derived EV bearing the B1-kinin receptor during vasculitis. In particular, neutrophil-derived EV bearing the B1 receptor were found docking to glomerular endothelial cells in kidney biopsies of patients with AAV. In vitro experiments showed that neutrophil-derived EV transfer functional B1-receptors to wild-type human embryonic kidney cells, which suggests a similar mechanism in vivo and could potentially promote kinin-associated inflammation. The observation that the main inhibitor of the kinin system, named the C1-inhibitor, is also involved in the inhibition of the release of EV enriched in B1-receptor has suggested a novel therapeutic target in vasculitis [93].

Prikryl et al. [94] recently performed proteomic profiling of urinary EV isolated from patients with AAV and renal involvement and in healthy controls. The study showed different levels of proteins potentially involved in AAV pathogenesis. As an example, they found a significantly decreased level of Golgi mannosidases, such as MAN1A1, both in urinary EV and in the whole urine in active AAV. Interestingly, MAN1A1 is involved in protein glycosylation, which is considered to be involved in autoimmune disease and T cell activation, supporting its role in the pathogenesis of AAV. Moreover, they showed different levels of proteins related to neutrophil activation and degranulation, platelet regulation and podocyte-associated proteins, to name a few [94].

Several studies have also shown that EV may contain enzymes linked to lipid metabolism. Indeed, a comparison between EV in active GPA and healthy controls showed an increased content of leukotriene (LT)B_4_ and 5-oxo-eicosatetraenoic acid (5-oxo-ETE) in EV from GPA patients. Moreover, neutrophils primed with GM-CSF, and stimulated with EV recovered from GPA patients, generate ROS and release dsDNA. Interestingly, in vitro-primed neutrophils were stimulated by LTB_4_ and 5-oxo-ETE, thus EV carrying lipid enzymes may contribute to AAV pathogenesis [95]. In a recent study, Surmiak et al. [96] stimulated human umbilical endothelial cells (HUVEC) with EV from anti-PR3-activated neutrophils and analyzed their miRNA and mRNA content. They found a miRNA/mRNA profile consistent with the release of proinflammatory cytokines, which may be involved in endothelial injury in vasculitis. The most increased cytokines were IL-8, IL-33, Dickkopf-related protein 1 (DKK-1), soluble interleukin (IL)-1 like receptor-1 (ST2) and angiopoietin-2. Interestingly this cytokine profile is similar to the circulating cytokine profile in GPA patients [96].

Recent studies have investigated the role of EV in mediating endothelial injury in MPA. EV can be taken up by glomerular endothelial cells in vitro and can increase the release of soluble cellular adhesion molecules (sICAM-1 and sVCAM-1) leading to the injury of the glomerular endothelial barrier. A sequencing analysis of EV miRNA cargo in MPA patients revealed a different miRNA profile in MPA patients. In particular, a correlation between miR-185-3p, miR-125a-3p and clinical parameters, such as BVAS and 24-h urine proteins, was reported. Thus, the EV-miRNA content has been proposed as a biomarker of renal involvement in MPA [97].

Circulating EV expressing MPO are elevated in AAV patients compared with healthy subjects. Interestingly, MPO+ EV expressing inflammatory biomarkers such as PTX3 and HMGB1 are associated with disease activity. Of interest, PTX3 is released by neutrophils during the inflammatory process, while HMGB1 may be associated with renal injury in AAV [98]. HMGB1 enhanced neutrophil activation and migration towards glomerular endothelial cells in the presence of ANCA, leading to glomerular cell injury and the release of TF-positive EV and endothelin-1, which is involved in the fibrogenesis [99].

The comparison of circulating EV expressing MPO in AAV patients and healthy controls reveals an increased expression of the complement components C3a and C5a on EV from AAV patients. Moreover, among AAV patients, C3a and C5a expression is higher in patients with active renal involvement compared to non-renal disease. Interestingly, the level of C3a and C5a expression on EV correlated with disease activity evaluated by BVAS [100].

Platelet-derived EV were also found to be increased in AAV patients, particularly in MPO-positive patients with active disease in whom EV also expressed higher levels of chemokines, adhesion molecules, growth and apoptotic factors. Moreover, EV level correlated with the disease activity and the renal involvement, with serum creatinine and glomerular histologic lesions [101].

Taken together, these results may provide insight into the role of EV in the pathogenesis of renal injury in AAV (Table 4).

## 5. Conclusions

Recent studies have demonstrated the relevance of EV in physiological and pathological processes [7]. Due to their role in immune system modulation, EV are considered crucial actors in the pathogenesis of autoimmune diseases [1].

Although current treatment options have improved their prognosis, autoimmune conditions remain rare diseases with high mortality and morbidity, particularly when the kidney is involved. Currently, kidney biopsy is the most important diagnostic and prognostic tool; however, recent investigations have focused on the identification and development of non-invasive biomarkers for early diagnosis and for the assessment of the disease activity.

Recently, the impact of EV in autoimmune disease with renal involvement has been investigated [3]. EV numbers and features seem to correlate with pathological processes. In addition, EV have been reported to enhance the autoimmune process and promote renal injury. In APS, the EV circulating level has been shown to reflect endothelial and platelet chronic activation and may correlate with thrombotic events [6,44,49]. Moreover, EV can actively contribute to SLE pathogenesis as an autoantigen source, part of the immune complexes, endothelial and leukocytes activation promoters [52,63]. Of interest, the galectin-3 binding protein (G3BP)+ EV and urinary high-mobility group box 1 molecule (HMGB1)+ EV seem to be involved in lupus nephritis pathogenesis [67,68,70]. Likewise, EV contribute to renal injury in STEC-HUS [79,80,81]. In ANCA-associated vasculitis, EV are involved in endothelial activation and correlate with disease activity [91,94]. Of interest, HMGB1+ EV and B1-receptor + EV can be directly involved in renal cell injury and have been proposed as a novel therapeutic target [93,98]. Moreover, miRNA carried by circulating EV can be investigated as novel diagnostic biomarkers for both lupus nephritis [72,74,76] and ANCA-associated vasculitis [97].

Encouraging discoveries on EV as diagnostic tools have been provided; however, being a recent research field, contradictory results reflecting the lack of standardized evaluation methods make their application in the clinical practice still challenging. Future studies should focus on defining standardized methods of EV collection and cargo evaluation. Of note, interesting observations have been reported in the research field, which need further investigation to be transferred in clinical settings.

However, the rareness of these diseases made it difficult to investigate the potential clinical impact of EV as diagnostic and prognostic tools in large clinical studies. For this reason, multicenter studies are needed to collect relevant data.

Despite these limitations, future studies on the role of EV in renal pathology should be pursued to better identify new targets in autoimmune diseases. Overall, the future challenge is to develop tools exploiting EV as diagnostic biomarkers, therapeutic targets or drug vectors for novel treatment options in autoimmune renal diseases.

## Figures and Tables

**Table 1:** Extracellular vesicles in antiphospholipid syndrome (APS).

Study	EV Biomarkers	Cellular Origin of EV	Study Findings	Reference
Štok, U.; et al.	CD8, CD44, CD133/1, CD62P	Platelets, endothelial cells, lymphocytes, antigen-presenting cells	EV increased in patients with thrombotic events EV reflect endothelial and platelet chronic activation	[6]
Chaturvedi, S.; et al., Breen, K.A.; et al.	CD41, CD61, CD51, CD105	Endothelial cells, platelets	EV increased in aPL+ patients EV reflect endothelial and platelet chronic activation	[44,45]
Chaturvedi, S.; et al., Willemze, R.; et al.	Tissue factor (TF)	Endothelial cells	TF + EV increased in APS TF activity increased in EV from aPL+ patients	[44,48]
Mobarrez, F.; et al.	β2GPI+		EV β2GPI+ reduced in SLE aPL+ Anti-β2GPI may bind to β2GPI expressed by EV	[19]
Campello, E.; et al.	Phosphatidylserine (PS), Endoglin, Tissue factor (TF)	Endothelial cells, platelets	PS+ EV, endoglin+ EV and endothelium-derived EV increased in 1st and 2nd trimester of pregnancy; TF+ EV and platelet-derived EV increased in 3rd trimester of pregnancy Correlation with thrombosis and systemic platelet and endothelial activation in obstetric APS	[49]

**Table 2:** Extracellular vesicles in systemic lupus erythematosus.

Study	EV Concentration	Cellular Origin of EV	EV Pathological Significance	Reference
Burbano, C.; et al.	Increased in SLE compared to healthy controls	platelet	Formation of immune complexes, source of nuclear antigens, correlation with disease activity	[52]
López, P.; et al.	Increased in SLE compared to healthy controls	platelet, monocyte, T lymphocyte	EV level correlated with: disease activity, glucocorticoid therapy, endothelial vasodilatation	[58]
Atehortúa, L.; et al.			Endothelial cell activation, endothelial injury,	[60]
Winberg, L.-K.; et al., Dieker, J.J.; et al., Rother, N.; et al.			In vitro stimulation of polymorphonuclear leukocytes with EV from SLE patients increased ROS production EV promote neutrophil activation and NETs production	[63,64,65]
Nielsen, C.T.; et al., Rasmussen, N.S.; et al.			IgG/galectin-3 binding protein (G3BP)+ EV are involved in the pathogenesis of lupus nephritis	[67,68]
Lu, J.; et al., Vanegas-García, A.; et al.	Urinary podocyte-derived EV increased in SLE	Urinary EV	Urinary podocyte-derived EV level correlated with systemic disease activity and renal injury Urinary EV high-mobility group box 1 molecule (HMGB1)+ were found to be higher in lupus nephritis	[69,70]
Felip, M.L.; et al., Solé, C.; et al., Navarro-Quiroz, E.; et al., Li, Y.; et al., Garcia-Vives, E.; et al.	EV derived miRNA		miR-21, miR-150, and miR-29c, miR-31, miR-107, and miR-135b-5p correlated with renal injury in lupus nephritis	[72,73,74,75,76]
Mobarrez, F.; et al.	EV containing mitochondrial molecules (mitoEV)		mitoEV were associated with disease activity, immune complex formation and renal damage	[77]

**Table 3:** Extracellular vesicles in thrombotic microangiopathies.

Disease	Study	Cellular Origin of EV	EV Biomarkers	EV Pathological Significance	Reference
**STEC-HUS**	Ståhl, A.-L.; et al., Arvidsson, I.; et al.	platelets, monocytes, neutrophils	Tissue factor, phosphatidylserine (PS), C3, C9	Promotion of thrombosis EV reflect complement activation	[22,78]
**STEC-HUS**	Varrone, E.; et al., Ståhl, A.-L.; et al., Johansson, K.; et al.		EV carrying Shiga toxin	Delivery system of Shiga toxin to the kidney involvement in renal cell injury	[79,80,81]
**TTP**	Tahmasbi, L.; et al., Jimenez, J.J.; et al.	platelets, endothelial cells	CD62E (E-selectin), VWF, intercellular adhesion molecule 1 (ICAM-1), platelet endothelial cell adhesion molecule (PECAM-1; CD31) and endoglin (CD105)	Pro-coagulant and pro-adhesive roles	[82,83]
**TTP**	Tati, R.; et al.	Endothelial cells	C3, C9	EV reflect complement activation	[84]

**Table 4:** Extracellular vesicles in ANCA-associated vasculitis.

Study	EV Biomarkers	EV Cellular Origin	Study Findings	Reference
Daniel, L.; et al.	proteinase 3 (PR3), myeloperoxidase (MPO)	EV released from primed neutrophils in vitro	EV can induce endothelial activation, ROS production, cytokines release	[91]
Brogan, P.A.; et al.		Platelets, neutrophils, endothelial cells	EV level increased in vasculitis Decrease of neutrophil-derived EV after treatment Endothelial-derived EV correlated with disease activity	[92]
Kahn, R.; et al.	B1 kinin receptor	Leukocytes	EV level increased in vasculitis Neutrophil-derived B1+ EV found on glomerular endothelial cells and renal injury	[93]
Prikryl, P.; et al.		Urinary EV	Proteomic EV profiling showed different regulation of proteins potentially involved in vasculitis pathogenesis	[94]
Surmiak, M.; et al.	leukotriene (LT)B4, 5-oxo-eicosatetraenoic acid (5-oxo-ETE)		EV enriched in LTB4 and 5-oxo-ETE in granulomatosis with polyangiitis	[95]
Wang, Y.; et al.			Sequencing analysis of EV miRNA cargo in microscopic polyangiitis identified a correlation between miR-185-3p, miR-125a-3p and both the clinical activity score and proteinuria	[97]
Manojlovic, M.; et al.	myeloperoxidase (MPO), PTX3, high mobility group box 1 (HMGB1)		PTX3+ and HMGB1+ EV correlated with disease activity HMGB1 potentially associated with renal injury	[98]
Antovic, A.; et al.	myeloperoxidase (MPO), C3a, C5a		MPO C3a+ and C5a+ EV increased in vasculitis, particularly in patients with renal involvement C3a and C5a expressed on EV correlated with disease activity	[100]
Miao, D.; et al.	chemokines, adhesion molecules, growth and apoptotic factors	Platelets	Increased EV in vasculitis EV correlate with disease activity and renal injury	[101]

## Data Availability

Not applicable.
